# Clinical and molecular characteristics of methicillin-resistant *Staphylococcus aureus* in bone and joint infection among children

**DOI:** 10.1186/s12941-023-00654-3

**Published:** 2023-11-22

**Authors:** Pan Fu, Yaxier Nijiati, Tingting Li, Xia Wu, Zixuan Wang, Jinlan Zhou, Chuanqing Wang, Bo Ning

**Affiliations:** 1 Department of Clinical Microbiology Laboratory, Children’s Hospital of Fudan University, National Children’s Medical Center, 399 Wanyuan Road, Shanghai, 201102 China; 2 Nosocomial Infection Control Department, Children’s Hospital of Fudan University, National Children’s Medical Center, Shanghai, China; 3 Orthopedics Department, Children’s Hospital of Fudan University, National Children’s Medical Center, 399 Wanyuan Road, Shanghai, 201102 China; 4 Department of Clinical Laboratory, Children’s Hospital of Fudan University, National Children’s Medical Center, Shanghai, China; 5 Department of Infectious Diseases, Children’s Hospital of Fudan University, National Children’s Medical Center, Shanghai, China; 6 Pediatric Intensive Care Unit, Children’s Hospital of Fudan University, National Children’s Medical Center, Shanghai, China

**Keywords:** Methicillin-resistant *Staphylococcus aureus*, Bone and joint infection, Panton–Valentine leukocidin, Virulence, Children

## Abstract

**Objective:**

To investigate the characteristics of Methicillin-Resistant *Staphylococcus aureus* (MRSA) in bone and joint infection (BJI) among children.

**Methods:**

A total of 338 patients diagnosed with BJI from 2013 to 2022 in Children’s Hospital of Fudan University were enrolled. Demographic information, microbiology culture results and laboratory findings, including white blood counts (WBC), C-reactive protein (CRP), procalcitonin (PCT), interleukin-6 (IL-6), and erythrocyte sedimentation rate (ESR) were collected and analyzed. MRSA was confirmed by antimicrobial susceptibility testing. Other MRSA-caused infections were randomly selected for comparison. Twenty-three virulence and antimicrobial resistance (AMR) genes were screened for MRSA strains. Multilocus sequence typing (MLST) and Staphylococcal protein A (spa) typing were performed using PCR amplification and sequencing.

**Results:**

Of the identified pathogens in BJI, MRSA accounted for 21.0% (47/224). Patients with BJI had high levels of initial CRP, white blood cell count (WBC) and IL-6. ST59 (43.9%) and t437 (37.6%) were the main MRSA subtypes isolated from the children. The major genotypes in BJI were ST59-t437 (29.8%) and ST22-t309 (14.9%), with high carriage of hemolysins including *hla* (94.4–100%), *hlb* (66.2–93.3%), and *hld* (100%). Notably, Panton–Valentine leukocidin (*pvl*) had a high prevalence (53.3%) in ST22-t309-MRSA. Other virulence genes including *tst*, *seg* and *sei* were more commonly detected in ST22-t309-MRSA (40.0–46.7%) than in ST59-t437-MRSA (4.2–9.9%). High-carriage AMR genes in MRSA included *aph(3ʹ)/III* (66.7–80%)*, ermB* (57.5–73.3%) and *ermC* (66.7–78.9%). MRSA presented high-resistance to erythromycin (52.0–100%) and clindamycin (48.0–92.5%), different genotypes displayed variation in their susceptibilities to antibiotics.

**Conclusions:**

The major MRSA genotype in BJI was ST59-t437, followed by ST22-t309, with a higher prevalence of the *pvl* gene. Continuous surveillance of *pvl*-positive ST22-t309-MRSA in pediatric BJI infections is thus required.

**Supplementary Information:**

The online version contains supplementary material available at 10.1186/s12941-023-00654-3.

## Introduction

Bacterial bone and joint infection (BJI) remains the most challenging and difficult-to-treat complication of orthopedic surgery in children. Previous studies showed that BJI incidence was much higher in children than in adults and more frequently affects children younger than 5 years of age [1, 2]. The most common bacteria causing pediatric BJI is *Staphylococcus aureus* (*S. aureus*), with a significant proportion of methicillin-resistant isolates [3]. Methicillin-resistant *S. aureus* (MRSA) is frequently resistant to many different classes of antibiotics and poses a serious problem because of its ability to evolve [4]. MRSA can cause a series of BJI, including osteomyelitis (bone infections), joint infections, and infections of implanted prosthetic devices (such as those used in knee replacement), etc. [3, 5].

MRSA strains have been shown to express a series of virulence genes including hemolysin (HL), staphylococcal enterotoxin (SE), exfoliative toxin (ET), Panton–Valentine leukocidin (PVL), and toxic shock syndrome toxin (TSST) [6]. PVL is a powerful staphylococcal exotoxin produced from the genetic material of a bacteriophage that infects *S. aureus*, making it more virulent [7]. It destroys the plasma membrane of polymorpho-nuclear cells and stimulates the release of inflammatory factors, including oxygen metabolites, interleukin-8, lysozymes, and histamine [8].

Compared to methicillin-sensitive *S. aureus* (MSSA), MRSA shows a multidrug-resistant pattern, not only for penicillin, methicillin, oxacillin and β-lactams, but also for variable antimicrobial classes including; macrolides, fluoroquinolones, aminoglycosides, tetracyclines, and chloramphenicol [6, 9, 10]. Notably, many studies have illustrated MRSA resistance to last-resort antibiotics, including vancomycin, linezolid and daptomycin [6, 11], which may become a looming threat to public health and further complicate the treatment of MRSA infection.

MRSA has spread worldwide, showing different clones of strain dissemination and evolving independently on different continents. Different MRSA clones have emerged in different regions, and ST239 and ST5 are the most prevalent MRSA clones [12]. The most prevalent MRSA in Asia is ST59, although it is not the epidemic clone in other parts of the world [13, 14]. ST59 is primarily linked to *S. aureus* protein A (spa) type t437 via the ST59-t437 linkage, which has fast growth ability, high survival rate resistance, high toxin secretion levels, and cytotoxicity [15]. Although there have been several reports of MRSA and pediatric BJI infections, systematic analyses of the molecular characteristics and epidemiology of MRSA in children with BJI are still lacking or limited worldwide [16, 17].

Therefore, MRSA is a serious concern for patients with BJI owing to its high virulence and antimicrobial resistance (AMR). Herein, we collected the MRSA strains which that pediatric BJI from 2013 to 2022. The clinical and molecular features, virulence genes, and AMR profiles of MRSA isolates in BJI were analyzed. This study reveals the epidemiology and molecular characteristics of MRSA-causing BJI in children. The major MRSA genotypes in BJI were ST59-t437 and ST22-t309, both of which harbored a series of virulence and AMR genes. ST22-t309-MRSA more commonly carried *pvl* and other virulence genes than ST59-t437-MRSA. Continuous surveillance of *pvl*-positive ST22-t309-MRSA in patients with BJI is of great significance.

## Methods

### Study design and enrollment of MRSA strains

A retrospective cohort study was performed on children admitted to the Children’s Hospital of Fudan University in Shanghai, China between January 2013 and December 2022 with a diagnosis of BJI. BJI includes septic arthritis, prosthetic joint infections, osteomyelitis, spinal infections, etc. [18]. Data collection was based on electronic medical records during hospitalization or clinic visits and data analysis was anonymous. Demographic information, laboratory findings, microbiological culture results, and antimicrobial susceptibility testing (AST) were collected and further analyzed.

MRSA was defined as *S. aureus* that showed resistance to methicillin or oxacillin based on AST results. All *S. aureus* strains isolated from the Children’s Hospital of Fudan University from 2013 to 2022 were analyzed using Whonet 5.0 (https://whonet.org/) to reveal the total MRSA percentage among children. MRSA strains which cause invasive infections other than BJI and respiratory infections, were randomly selected for comparison. Invasive infection was defined as the isolation of a bacterial organism from normally sterile body fluids, such as blood, cerebrospinal fluid, pleural fluid, pericardial fluid, or deep tissue abscesses [19].

### Antimicrobial susceptibility testing (AST)

The AST was performed on a VITEK 2 compact system using AST-GN13 cards (bioMérieux). The KB method is additionally performed. The results were interpreted according to the criteria of the Clinical and Laboratory Standards Institute (CLSI) 2022 breakpoints [20]. Antibiotics included: penicillin (PEN), oxacillin (OXA), macrolides including ERY (erythromycin) and CLI (clindamycin), tetracycline (TET), fluoroquinolones including levofloxacin (LEV) and ciprofloxacin (CIP), trimethoprim/sulfamethoxazole (SXT), rifampicin (RIF), fosfomycin (FOS), gentamicin (GEN), linezolid (LZD), vancomycin (VAN), teicoplanin (TEC), and tigecycline (TGC).

### Multilocus sequence typing (MLST)

The total DNA of MRSA isolates was prepared and MLST was performed with housekeeping genes of *S. aureus* (*arcC, aroE, glpF, gmk, pta, tpi,* and *yqiL*) based on the protocol described on the MLST database (https://pubmlst.org/) according to the method reported by Mark Enright [21]. The sequence type (ST) of each isolate was assigned based on the MLST database. A phylogenetic tree was generated using GrapeTree (https://github.com/achtman-lab/GrapeTree) based on the MLST profiles.

### Staphylococcal protein A (Spa) typing

Spa typing was performed as previously described [22]. Spa primers F: 5ʹ-AGACGATCCTTCGGTGAGC-3ʹ and R: 5ʹCAGCAGTAGTGCCGTTTG-3ʹ were used in this study. Repeats were assigned a numerical code and the spa-type is deduced from the order of specific repeats. The Ridom Spa Server database (http://www.spaserver.ridom.de/) was used to assign edited sequences to particular spa types.

### Detection of virulence-associated genes and AMR genes

Sixteen toxin genes with reported contributions to virulence were selected, including hemolysin toxins (*hla, hlb, hld*), enterotoxins genes (*sea, seb, sec, sed, see, seg, seh, sei, sej*), exfoliative toxin genes (*eta* and *etb*), toxic shock syndrome toxin (TSST-1) toxin gene (*tst*), and *pvl*. Seven AMR genes including *mecA*, aminoglycoside-resistance genes (*aph(3ʹ)/III* and *aac(6ʹ)/aph(2″)*), erythromycin-resistance genes (*ermA*, *ermB* and *ermC*) and tetracycline-resistance gene *tetM*. All MRSA isolates were screened for the presence of those genes by PCR amplification and sequencing as previously described [23, 24].

### Statistical analysis

Statistical analyses were performed using GraphPad Prism 8.0 (GraphPad Software, Inc., San Diego, California, USA). Data were analyzed using the χ2 test or Fisher’s exact test, as appropriate. A *p*-value of < 0.05 was considered statistically significant.

## Results

### MRSA strains in pediatric BJI

From January 2013 to December 2022, 338 patients were diagnosed with BJI at the Children’s Hospital of Fudan University, of which 224 (69.2%) had a microbiological diagnosis. *S. aureus* was the most frequently identified pathogen (155, 69.2%), followed by *Pseudomonas aeruginosa* (41, 18.3%). A total of 47 MRSA strains were identified, accounting for 21.0% of the pathogens in pediatric BJI.

MRSA strains that caused other invasive infections (67 strains) and respiratory infections (75 strains) were randomly selected during the same period for comparison.

### AMR profiles and characteristics of MRSA in BJI

**Fig. 1:**
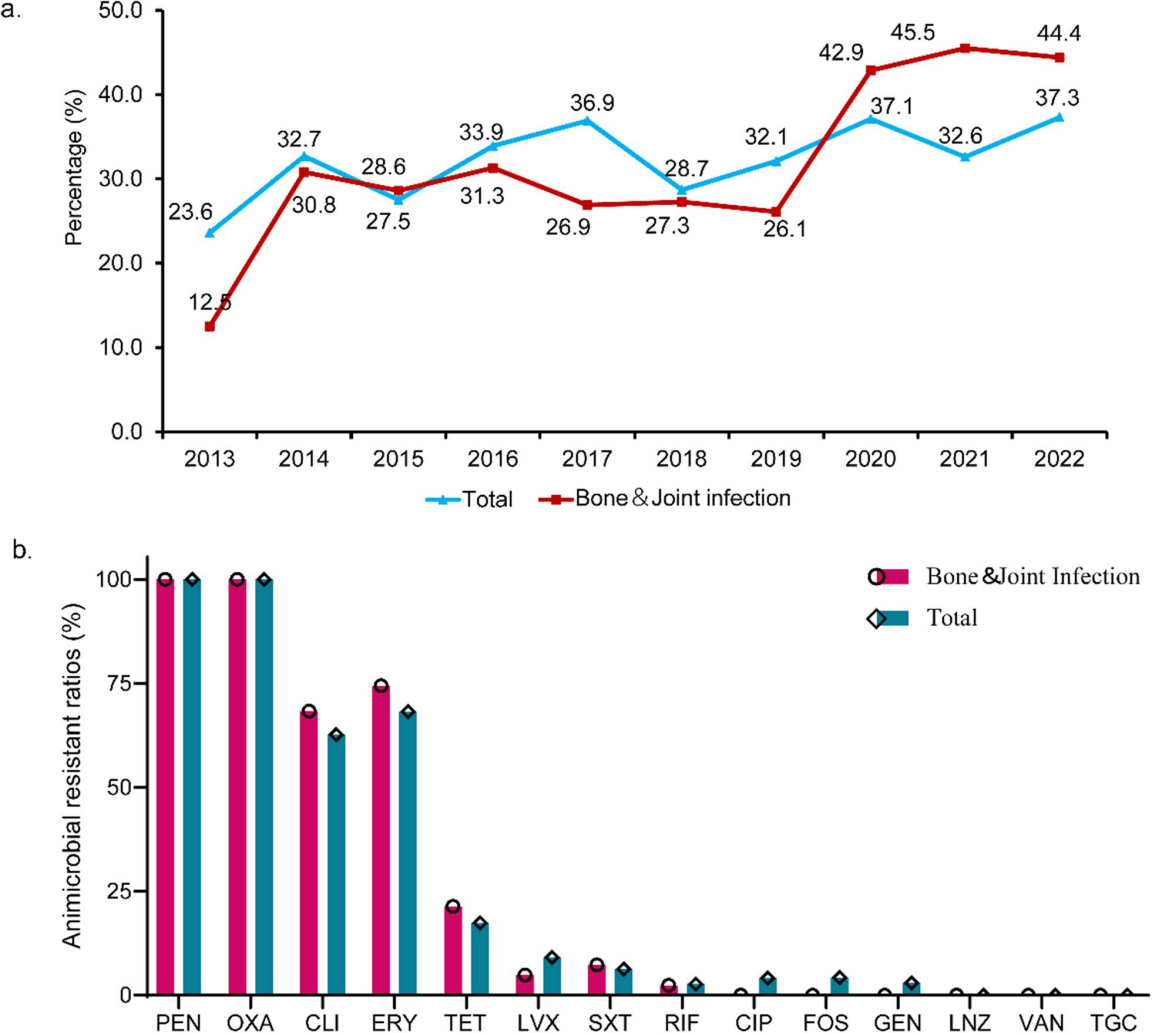
Percentage and AMR profiles of MRSA isolate from patients with BJI and total cases from 2013 to 2022. **a** MRSA ratios in BJI and total infection in children, 2013–2022. **b** AMR profiles of MRSA strains isolated from patients with BJI and total cases during 2013 to 202

The total MRSA ratio is predicted to range from 23.6% in 2013 to 37.3% in 2022. Remarkably, MRSA isolated from patients with BJI increased from 12.5% in 2013 to 44.4% in 2022. The MRSA ratios in BJI which were lower than or similar to the total levels from 2013 to 2019, exhibited a noticeable increase from 2020 to 2022. MRSA isolated from patients with BJI and all cases exhibited high resistance to CLI (62.7–68.3%) and ERY (68.2–74.4%) and lower resistance to TCY (17.3–21.4%). Resistance to LVX, SXT, RIF, CIP, FOS and GEN was rare and no strains presented resistance to LNZ, VAN, TEC or TGC (Fig. 1).

A total of 189 patients with BJI (47 cases), invasive infection (67 cases) and respiratory infection (75 cases) were enrolled. The average age of patients with BJI was 5.0 ± 0.6 years. Patients with BJI present with higher levels of inflammatory factors, including initial white blood cell counts (WBC), C-reactive protein (CRP), procalcitonin (PCT), interleukin-6 (IL-6), and erythrocyte sedimentation rate (ESR). Patients with BJI presented a significant higher ESR ((67.9 ± 8.6) mm/h) than other infections (p < 0.05, Table 1).

**Table 1:** Clinical characteristics and laboratory testing of different type of MRSA infections in children

Clinical information		Bone & joint infection (n = 47)	Invasive infection (n = 67)	Respiratory infection (n = 75)	p-value
Gender	Male (n, %)	31 (66.0%)	37 (55.2%)	36 (48.0%)	
	Female (n, %)	16 (34.0%)	30 (44.8%)	39 (52.0%)	
Age (year)		5.0 ± 0.6	3.3 ± 0.5	2.7 ± 0.4	
Laboratory testing	WBC (× 10^9^/L)	15.9 ± 1.4	14.2 ± 0.9	12.1 ± 0.7	0.016
	CRP (mg/L)	55.4 ± 8.3	47.2 ± 5.9	21.1 ± 2.9	< 0.001
	PCT (ng/L)	1.8 ± 0.6	1.5 ± 0.5	1.2 ± 0.7	ns
	IL-6 (pg/mL)	129.2 ± 48.5	121.4 ± 28.4	98.9 ± 32.1	0.031
	ESR (mm/h)	67.9 ± 8.6	36.4 ± 9.4	35.2 ± 10.7	0.002

*WBC* White blood cell counts, *CRP* C-reactive protein, *PCT* procalcitonin, *IL-6* interleukin-6, percentage of neutrophil (N%) and *ESR* Erythrocyte sedimentation rate

### Characteristics and profiles of MLST and spa subtypes of MRSA in BJI

**Fig. 2:**
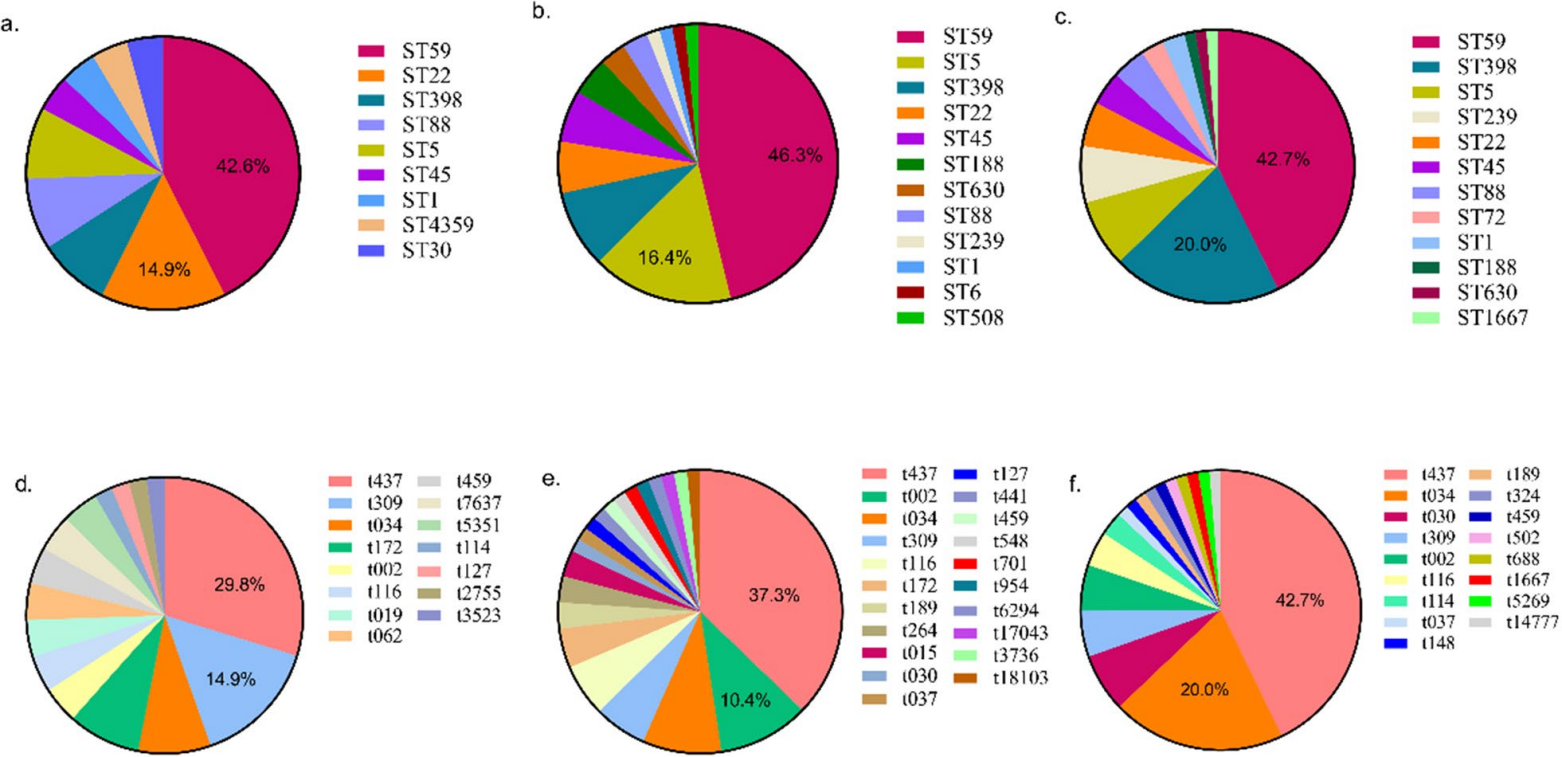
MLST and spa subtypes of MRSA strains isolated from pediatric patients with BJI, invasive infection, and respiratory infection. MLST types of MRSA strains in BJI (**a**), invasive infection (**b**), and respiratory infection (**c**). spa types of MRSA strains in BJI (**d**), invasive infection (**e**), and respiratory infection (**f**)

As shown in Fig. 2, MRSA isolates were classified into 9 STs and 15 spa types in BJI, 12 STs and 21 spa types in invasive infections, and 12 STs and 17 spa types in respiratory infections, respectively. ST59 (43.9%) and t437 (37.6%) were the predominant MRSA subtypes. Noticeably, ST22 was ranked second and accounted for 14.9% of BJI cases, whereas ST5 and ST398 were the second most common types of invasive infections (16.4%) and respiratory infections (20.0%), respectively.

**Fig. 3:**
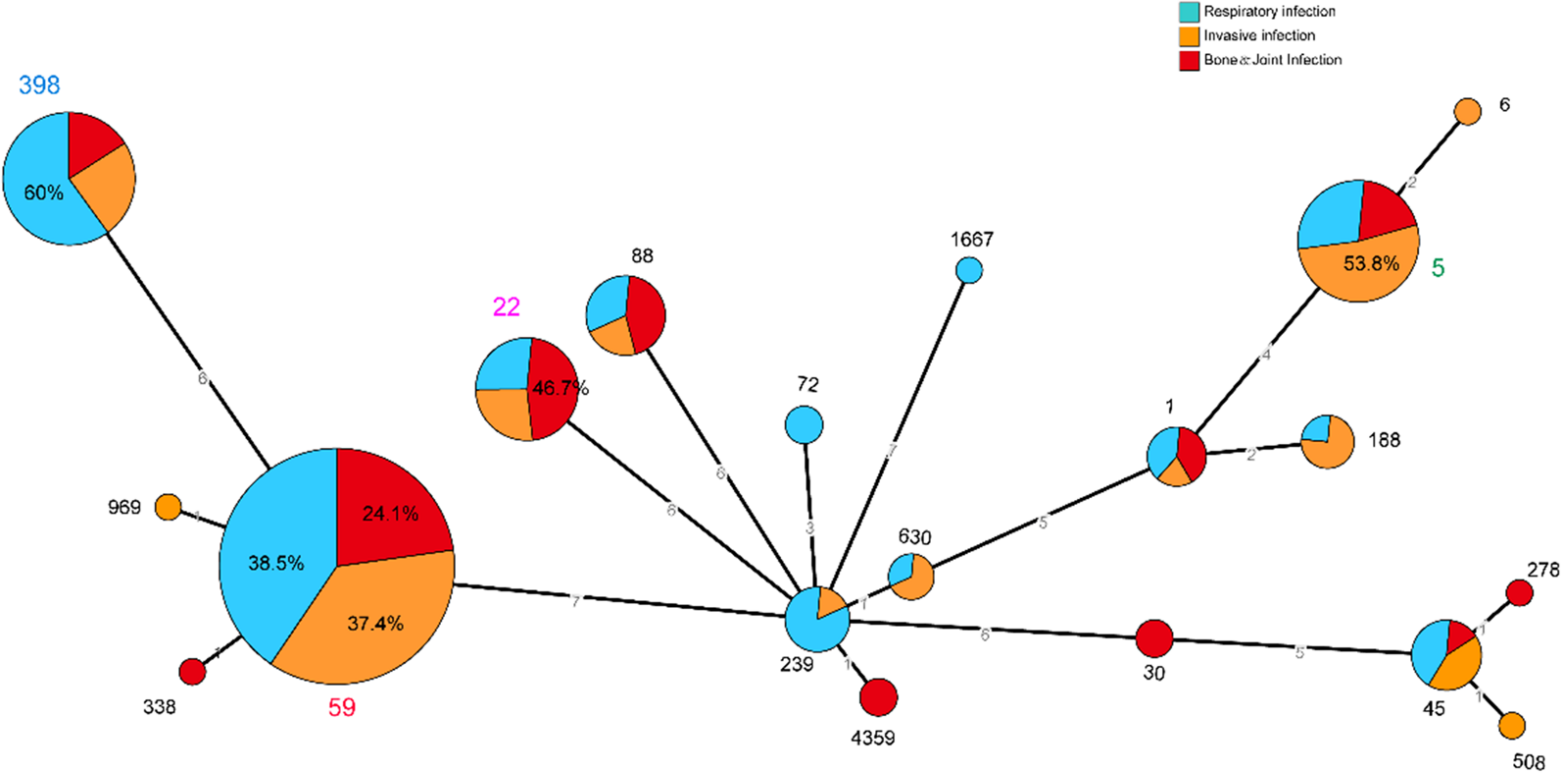
Minimum-spanning tree of MRSA isolates based on MLST types in BJI, invasive infection and respiratory infection. Circle size indicates the number of each ST type. Differences in the length of the lines linking two circles indicate differences in the number (marked on the line) of alleles between the two linked ST types

The main MLST types of all MRSA strains were ST59 (43.9%), ST398 (13.2%), ST22 (11.1%) and ST5 (7.9%). Microevolutionary analysis of STs revealed that ST59, ST398, ST22 and ST5 were heterogeneous in different clusters. There were 24.1%, 37.4%, and 38.5% of ST59-MRSA isolated from BJI, invasive infection and respiratory infection, respectively. ST22-MRSA was mainly isolated from BJI (46.7%), whereas ST398 and ST5 were mainly detected in respiratory infections (60%) and invasive infections (53.8%) (Fig. 3).

**Fig. 4:**
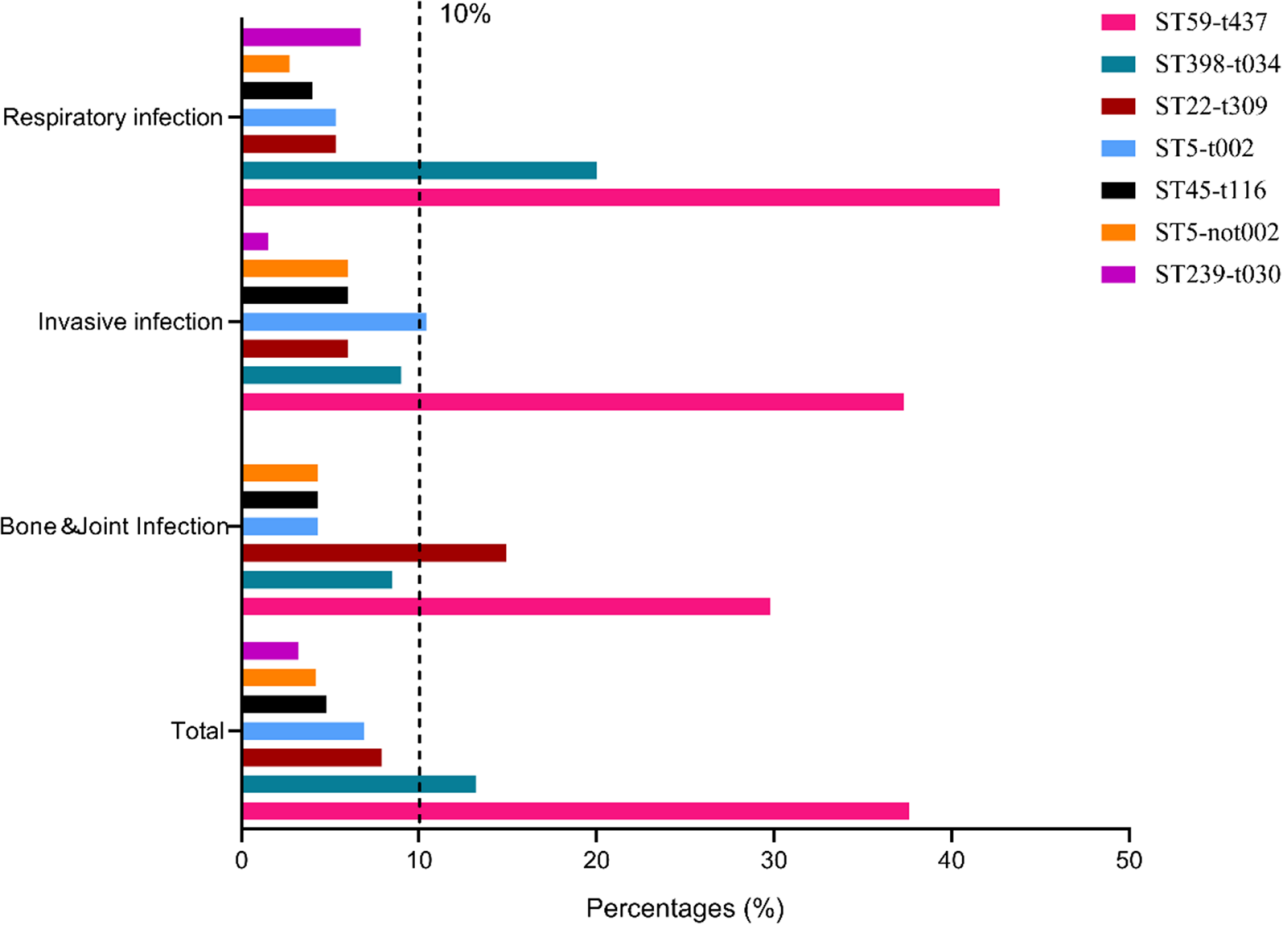
The major MRSA genotypes in total case, BJI, invasive infection and respiratory infection

Figure 4 shows the major genotypes including ST59-t437 (37.6%), ST398-t034 (13.2%), ST22-t309 (7.9%) and ST5-t002 (6.9%). ST59-t437 was the primary genotype in BJI (29.8%), invasive infections (37.3%), and respiratory infections (42.7%). ST22-t309, which ranked second (14.9%) in BJI, accounted for only 6.0% of invasive infections and 5.3% of respiratory infections. However, ST5-t002 and ST398-t034 which were rarely (4.3% and 8.5%) isolated from BJI, ranked second among invasive (10.4%) and respiratory infections (20.0%).

### Expression of virulence genes and antimicrobial resistant (AMR) genes in different MRSA genotypes

**Fig. 5:**
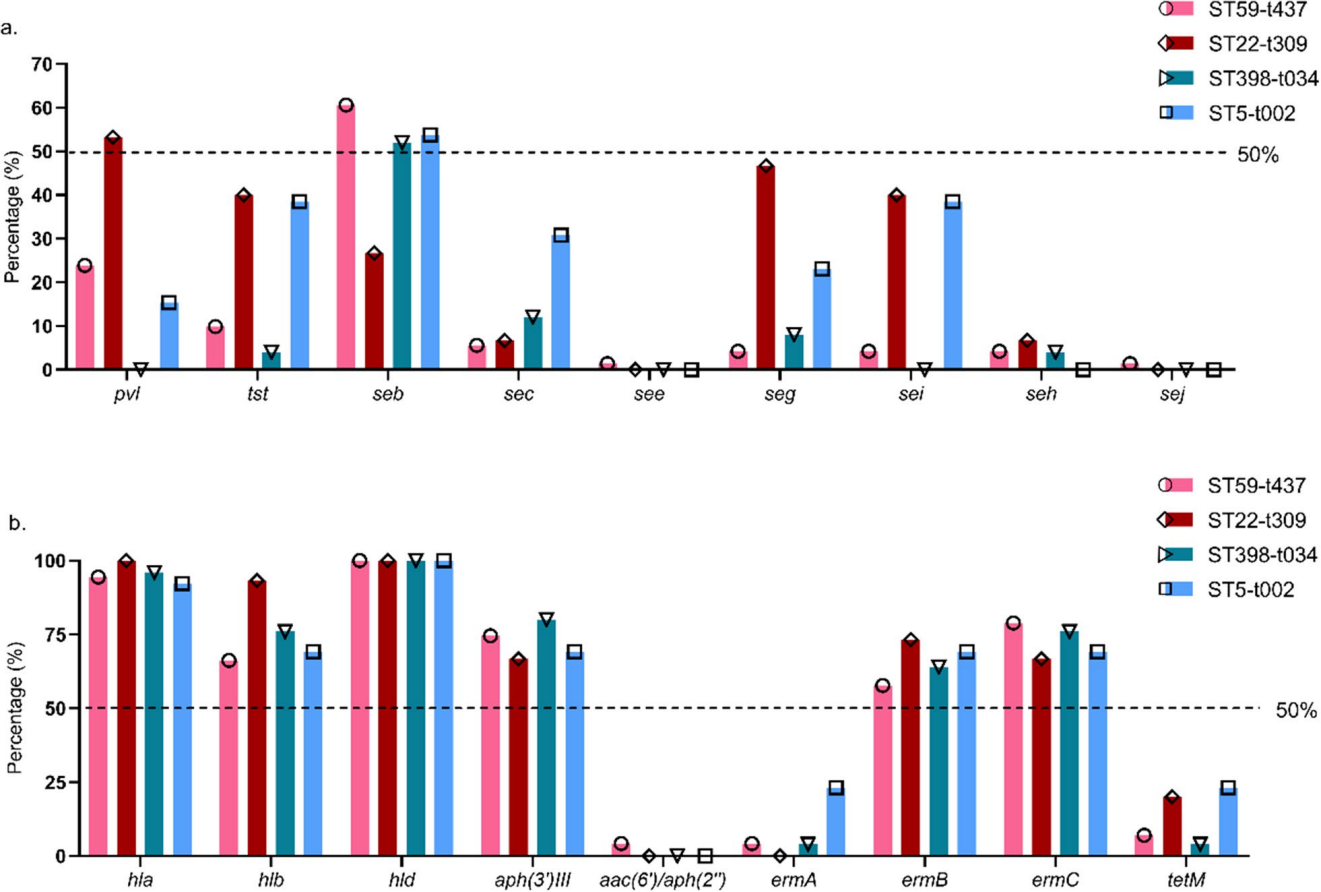
Expression of virulence genes and antimicrobial resistant genes in different MRSA genotypes

All MRSA isolates expressed *mecA*; however, none carried *sea*, *sed*, *eta* and *etb* genes. Figure 5 shows a very high carriage of hemolysins, including *hla* (92.3–100%), *hlb* (66.2–93.3%), and *hld* (100%) in different MRSA genotypes. Notably, *pvl* was exclusively highly carried (53.3%) by ST22-t309 and had much lower expression in other genotypes (0–23.9%, p < 0.01). Other virulence factors including *tst*, *seg,* and *sei,* were moderately expressed in ST22-t309-MRSA (40.0–46.7%), while *seb* was detected less in ST22-t309 (26.7%). (Fig. 5a and b).

Figure 5b depicts the AMR gene expression in different MRSA genotypes. The aminoglycoside resistance gene *aph(3ʹ)/III* was highly prevalent (66.7–80.0%), whereas *aac(6ʹ)/aph(2″)* was rarely detected in MRSA strains (0–4.2%). For erythromycin and tetracycline resistance genes, *ermB* and *ermC* were highly (57.7–73.3% and 66.7–78.9%) detected, but *ermA* and *tetM* were lowly (0–23.1% and 4.0–23.1%) detected from MRSA strains.

### Antimicrobial resistance profiles of different MRSA subtypes

All MRSA strains showed 100% resistance to PEN and OXA, and none (0%) were resistant to LZD, VAN, TEC or TGC. The antimicrobial susceptibility testing revealed high resistance rates to ERY and CLI in the MRSA strains. A few MRSA strains were resistant to SXT (0–4.0%), RIF (0–6.7%), CIP (0–1.4%), and GEN (0–1.4%). Different MRSA genotypes show variations in their susceptibility to antibiotics. For example, resistance to CLI and ERY was much lower in ST398-t034-MRSA (48.0% and 52.0%, respectively; p < 0.05), whereas resistance to LEV and FOS was significantly higher (53.8% and 38.5%, respectively; p < 0.05) in ST5-t002-MRSA. (Additional file 1: Table S1).

## Discussion

As an important pathogenic bacterium in human, MRSA is considered more severe in BJI, leading to greater morbidity than other pathogens [25]. In this study, MRSA accounted for 21.0% of the identified pathogens in pediatric BJI, presenting high resistance to PEN, OXA, ERY, and CLI. The MRSA ratio in BJI increased from 12.5% in 2013 to 44.4% in 2022. Major MRSA genotypes in pediatric BJI were ST59-t437 and ST22-t309. Notably, *pvl* was highly expressed in ST22-t309-MRSA strains. ST59-t437, the dominant MRSA genotype in BJI, reflects that observed in other populations studied in China. However, ST22-t309 that was prevalent in MSSA strains in China, has been ranked as the second most prevalent MRSA genotype in children with BJI [26].

ESR and CRP play important roles in first-line screening tests for BJI such as periprosthetic joint infections (PJI), PCT has been found to be useful in the post-operative setting where ESR and CRP remain elevated [27, 28]. In this study, BJI patients with MRSA infection had high levels of inflammatory factors including initial CRP, WBC count, PCT, IL-6 and ESR, of which only ESR was significantly higher in BJI, indicating its potential role as a specific serum biomarker for BJI.

Methicillin resistance is encoded by the *mecA* gene, located on a mobile genetic element called the staphylococcal cassette chromosome mec (SCCmec). MRSA evolved from MSSA strains via the acquisition of SCCmec [29]. All MRSA isolates in this study carried *mecA*. Previous study in China showed that ST59-t437 was the most prevalent MRSA genotype, whereas ST22-t309 more frequently detected in MSSA [26, 30]. Our findings demonstrate that ST59-t437 was the major MRSA genotype isolated from children, whereas ST22-t309, ST5-t002 and ST398-t034 ranked second in BJI, other invasive infections and respiratory infections, respectively.

Previous study showed that the prevalence of ST59-t437-MRSA in China increased from 3.6% in 2010–2011 to 9.1% in 2013, and then further reached to 15.1% in 2016 [31, 32]. ST59-t437-MRSA is considered to have a greater fitness advantage than other genotypes [31]. Together, ST59-t435 accounted for 37.6% of all MRSA strains in this study, revealing that MRSA diversity was dynamically evolving and ST59-t435-MRSA had the potential to spread among children.

ST22 is a successful MRSA lineage identified in hospitals, with a strong capacity to replace other formerly epidemic MRSA clones [33, 34]. Although ST22-MRSA has spread across many countries, especially in Australia and the United Kingdom, it remains sporadic in China [35]. Phylogenetic reconstruction and time estimation have suggested that ST22 in China emerged around 2006; ST22-MRSA evolved from native ST22-MSSA and displayed higher virulence than ST22-MSSA predecessors [35]. Our study showed that ST22, which is closely linked to t309, ranked second (14.9%) among MRSA strains isolated from patients with BJI, revealing the potential spread of this clone in BJI.

*Staphylococcus aureus* strains express a series of virulence genes, among which PVL causes leukocyte destruction and necrotizing pneumonia, an aggressive condition that kills up to 75% of patients [6, 36]. ST22 *pvl*-producing *S. aureus* is emerging globally [37]. Besides high carriage of *pvl*, ST22-t309-MRSA also displayed a series of virulence factors including hemolysin toxins (93.3–100%), *tst* (40%), *seg* (46.7%) and *sei* (40%), and AMR genes including *aph(3ʹ)/III, ermB* and *ermC* (66.7–73.3%). Therefore, our study reveals the emerging potential spread of *pvl*-positive ST22-t309-MRSA in patients with BJI.

The MRSA isolated from children in this study showed very high resistance to PEN, OXA, CLI and ERY, and different MRSA genotypes displayed variation in their susceptibility to a series of antibiotics. As the most predominant lineages in BJI, both ST59-t437 and ST22-t309 showed very high resistance to ERY and CLI, indicating that macrolides are no longer suitable for treating MRSA infections in patients with BJI.

In conclusion, we systematically analyzed the clinical and molecular characteristics of MRSA strains isolated from children with BJI. The major genotypes of the MRSA strains isolated from patients with BJI are ST59-t437 and ST22-t309, both of which carry a series of virulence and AMR genes. The *pvl* gene is more frequently detected in ST22-t309-MRSA; thus, continuous surveillance of MRSA molecular epidemiology among patients with BJI is required.

### Supplementary Information

**Additional file 1: Table S1.** Antimicrobial resistance ratios of different MRSA genotypes (%).

## Data Availability

Not applicable.
