# Supplementary material for: Clinical and molecular characteristics of methicillin-resistant Staphylococcus aureus in bone and joint infection among children
Source: Ann Clin Microbiol Antimicrob. 2023 Nov 22;22:104. doi: 10.1186/s12941-023-00654-3 (PMC10666310; doi:10.1186/s12941-023-00654-3)
Supplement: Supplementary file 1 — Additional file 1: Table S1. Antimicrobial resistance ratios of different MRSA genotypes (%). [file 12941_2023_654_MOESM1_ESM.docx]

| Table S1. Antimicrobial resistance ratios of different MRSA genotypes (%) | | | | | |
| --- | --- | --- | --- | --- | --- |
| Antibiotics | ST59-t437 (n=71) | ST398-t034 (n=25) | ST22-t309 (n=15) | ST5-t002 (n=13) | P-value |
| PEN | 100.0% | 100.0% | 100.0% | 100.0% | ns |
| OXA | 100.0% | 100.0% | 100.0% | 100.0% | ns |
| ERY | 83.1% | 52.0% | 80.0% | 100.0% | 0.002 |
| CLI | 81.7% | 48.0% | 73.3% | 92.5% | 0.003 |
| LVX | 0.0% | 0.0% | 6.7% | 53.8% | <0.001 |
| SXT | 1.4% | 4.0% | 0.0% | 0.0% | ns |
| RIF | 1.4% | 4.0% | 6.7% | 0.0% | ns |
| CIP | 1.4% | 0.0% | 0.0% | 0.0% | ns |
| FOS | 0.0% | 0.0% | 6.7% | 38.5% | <0.001 |
| GEN | 1.4% | 0.0% | 0.0% | 0.0% | ns |
| LNZ | 0.0% | 0.0% | 0.0% | 0.0% | ns |
| VAN | 0.0% | 0.0% | 0.0% | 0.0% | ns |
| TEC | 0.0% | 0.0% | 0.0% | 0.0% | ns |
| TGC | 0.0% | 0.0% | 0.0% | 0.0% | ns |

ns: no significance
